# Editorial: Return to sport training and testing

**DOI:** 10.3389/fspor.2024.1523274

**Published:** 2024-11-26

**Authors:** Sylvia Lin, Mathew Failla, Ryan Zarzycki

**Affiliations:** ^1^ Program in Physical Therapy, Washington University in St. Louis, St. Louis, MO, United States; ^2^ College of Nursing and Health Sciences, University of Vermont, Burlington, VT, United States; ^3^ Arcadia University, Glenside, PA, United States

**Keywords:** return to sport (RTS), return to sport criteria, injury, re-injury, re-injury risk

**Editorial on the Research Topic**
Return to sport training and testing

Return to sport is a primary goal for injured athletes and a central focus of their rehabilitation. Despite this, late phase rehabilitation programs and return to sport criteria remain unstandardized. Return to sport rates are lower than expected, and re-injury risk remains high for many athletes. While return to sport evidence is mounting in several areas, debate still exists amongst rehabilitation professionals around how to best achieve a return to prior-level of performance and to minimize re-injury risk. Many of these debates are a consequence of our lack of robust evidence related to key return to sport questions:

• What is the ideal combination of objective measures to demonstrate physical readiness to return to sport?
• How do we mitigate secondary and tertiary risk?
• When and how do we address psychological readiness to return to sport?
• How do we overcome gaps in healthcare systems after formal rehabilitation has ended but before return to sport has occurred?

In this Research Topic, we explore five different facets of this topic through the perspective of five unique manuscripts, including three original research articles.

Wang et al. discuss leveraging telehealth as an effective, cost-effective way to address acute shoulder pain in a young semi-professional tennis player. During a virtual training session, the athlete's guardian was instructed how to perform basic PT assessments and perform them with the athlete, so that the clinician could diagnose and treat the suspected condition. While high-quality evidence regarding the effectiveness of telehealth compared to in-person care is currently lacking, the case study by Wang et al. provides a template for clinicians to use telehealth effectively with young athletes who have poor access to healthcare.

Armitage et al. focus on summarizing the current state of literature regarding football injury prevalence, etiology, as well as on-field rehabilitation and return to play frameworks. They provide suggestions for future research based on knowledge and implementation gaps.

Huynh et al. examine the specific physical demands of Division I collegiate basketball players through video-based motion analysis, comparing by sex and playing position. Significant differences in activity requirements were found in both categories, which the authors propose should be considered during rehabilitation and return to sport decision-making.

Becker et al. examine the effect of fatigue on athletes’ knee stability during a single drop landing. Given the conflict in existing literature regarding fatigue and knee kinematics, this study sought to induce cardiovascular fatigue comparable to sporting activity. The authors propose consideration of fatigue in injury prevention, during rehabilitation, and return to sport testing.

Finally, Weber et al. examined the relationship between common hop tests used in return to sport testing and movement quality. The authors found no relationship between hop test symmetry and the “Quality First” assessment. These findings highlight the importance of using qualitative assessments of movement quality in addition to traditional hop tests.

Given the positive engagement, we believe that this special issue on *Return to sport training and testing* has made an important contribution to the literature in areas of need. We hope these articles stimulate ideas and generate further discussion and research on this multifaceted and complex, evolving issue.

